# ALKBH4 Depletion in Mice Leads to Spermatogenic Defects

**DOI:** 10.1371/journal.pone.0105113

**Published:** 2014-08-25

**Authors:** Anja Nilsen, Markus Fusser, Gareth Greggains, Peter Fedorcsak, Arne Klungland

**Affiliations:** 1 Institute of Medical Microbiology, Oslo University Hospital, Rikshospitalet, University of Oslo, Oslo, Norway; 2 Section for Reproductive Medicine, Department of Gynecology, Oslo University Hospital, Rikshospitalet, Oslo, Norway; 3 Institute of Basic Medical Sciences, University of Oslo, Oslo, Norway; University of Science and Technology of China, China

## Abstract

ALKBH4, an AlkB homologue in the 2-oxoglutarate and Fe^2+^ dependent hydroxylase family, has previously been shown to regulate the level of monomethylated lysine-84 in actin and thereby indirectly influences the ability of non-muscular myosin II to bind actin filaments. ALKBH4 modulates fundamental processes including cytokinesis and cell motility, and its depletion is lethal during early preimplantation embryo stage. The aim of this study was to investigate the effect of ALKBH4 deficiency in a physiological context, using inducible *Alkbh4* knockout mice. Here, we report that ALKBH4 is essential for the development of spermatocytes during the prophase of meiosis, and that ALKBH4 depletion leads to insufficient establishment of the synaptonemal complex. We also show that ALKBH4 is localized in nucleolar structures of Sertoli cells, spermatogonia and primary spermatocytes.

## Introduction

The nine mammalian homologues of the *E. coli* DNA demethylase AlkB, ALKBH1 through to ALKBH8 and FTO, display diverse enzymatic activities. ALKBH2-3,-5,-8 and FTO are oxidative demethylases acting on methyl groups of nucleic acids [1]–[5], whereas ALKBH1 exhibits hydroxylase activity on methylated histone H2A [6].

We have recently described that ALKBH4 interacts with a mono-methylated lysine (K84me1) in actin [7]. Depletion of ALKBH4 both in primary and in transformed cell cultures resulted in increased levels of actin-K84me1, suggesting that ALKBH4 is a K84me1-actin demethylase. Notably, non-muscular myosin II is unable to interact with actin-K84me1, and consequently, depletion of ALKBH4 results in major cellular defects, including failure of cytokinesis, reduced cell migration and increased cell death [7]. Accordingly, we have shown that loss of ALKBH4 is lethal during early embryonic development. ALKBH4 is expressed both in the cytoplasm and in the nucleus of interphase cells, and accumulates in the midbody during cytokinesis [7], [8]. Based on protein interaction studies and visualization of ectopically expressed ALKBH4 in nucleoli in somatic cells, a role for ALKBH4 in gene transcription has also been proposed [9].

Male germ cells undergo DNA synthesis, homologous chromosome pairing and recombination during the prophase of meiosis. Chromosomal pairing and homologous recombination are facilitated by the synaptonemal complex (SC), a zipper-like protein structure that assembles between bivalents during meiotic prophase [10], [11]. A diverse range of errors that can occur during the meiotic prophase are monitored by multiple molecular checkpoints (reviewed in [12]).

The chromosomes are actively repositioned during the meiotic prophase. Before zygotene, the telomeres associate with the nuclear envelope (NE) in meiosis-specific complexes. During zygotene, these ends cluster into a localized area of the NE (the bouquet) while, at early pachytene, the ends redistribute throughout the nuclear periphery [13]–[16]. In the diplonema, separation of the homologous chromosomes begins, which is completed during diakinesis. The spermatocyte then undergoes two meiotic divisions to form spermatids, which go through a differentiation phase called spermiogenesis [17]. The somatic Sertoli cells provide the structural support for the germinal epithelium and the physiological environment for spermatocyte development. Germ cells are attached to Sertoli cells by actin-based adherence junctions (ectoplasmic specialization, ES), which assist in the translocation of early spermatocytes towards the lumen of seminiferous tubuli [18].

Concluding the cytokinesis during mitosis, the midbody structure is abscised to separate the two cells [19]. During meiosis, however, dividing germ cells do not abscise the midbody and stay connected through a stable syncytium linking the cytoplasm of generations of daughter cells [20].

To investigate the physiological role of ALKBH4 and circumvent embryonic lethality of *Alkbh4* deletion [7], we depleted ALKBH4 in mice using a tamoxifen-inducible *Alkbh4^L/L^ CreEsr* strain. Here, we report that ALKBH4 depletion leads to a marked loss of male germ cells during meiotic prophase and disorganization of the synaptonemal complex. Moreover, we describe the nuclear localization pattern of ALKBH4 in mitotic and premeiotic male germ cells in addition to Sertoli cells.

## Results

### Characterization of *Alkbh4^Δ/Δ^* mice

**Figure 1:**
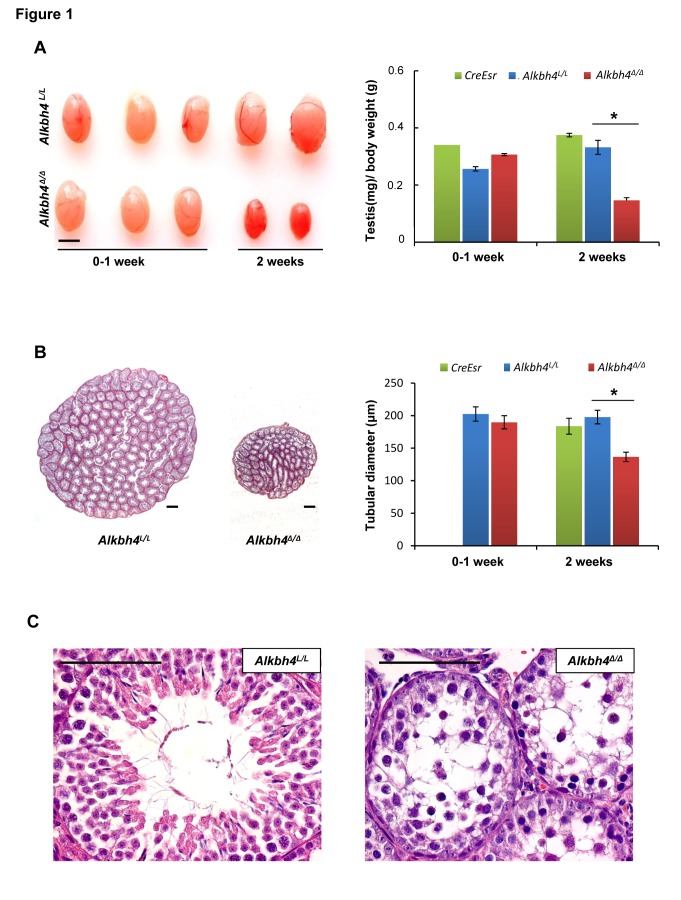
Loss of Alkbh4 reduces the size of testes and the diameter of seminiferious tubuli. The phenotype of testis of tamoxifen-treated mice with genotypes *Alkbh4^L/L^* (Control), CreEsr (Control) and *Alkbh4 ^Δ/Δ^*. A Left panel, representative testes from control and *Alkbh4 ^Δ/Δ^* mice after tamoxifen treatment for one week or without treatment (0–1 week), and after deletion of *Alkbh4* with treatment for 2 weeks. Scale bar, 0.5 cm. Right panel, average testis/body weight ratio of 4–5 weeks old mice before and after 1 week of treatment with tamoxifen (0–1 week), and 6 weeks old mice treated with tamoxifen for 2 weeks (0–1 week: *Alkbh4^L/L^*, n = 3; CreEsr, n = 1; *Alkbh4^Δ/Δ^*, n = 3. 2 weeks: *Alkbh4^L/L^*, n = 5; *CreEsr*, n = 6; *Alkbh4 ^Δ/Δ^*, n = 6). Data are expressed as means ± SEM. B Left panel, histological overview of representative control and *Alkbh4 ^Δ/Δ^* testis after 2 weeks of tamoxifen induction (HE stain, scale bar, 20 µm). Right panel, decreased diameter of seminiferous tubuli in *Alkbh4 ^Δ/Δ^* mice after 2 weeks tamoxifen treatment compared to control mice (n = 100 tubuli/animal, 2 animals/genotype/time point. 0–1 week CreEsr N/A). Data are expressed as means ± SEM. **C** High magnification view (scale bar, 20 µm) of testis section in B. Statistical analysis was performed using a non-paired, two-tailed Student's test: *p<0.05.

Based on our previous study, which shows the requirement of ALKBH4 in cell proliferation and migration, we wanted to study the effect of ALKBH4 depletion in developing juvenile mice. Inducible *Alkbh4^L/L^ CreEsr* knockout mice have been described previously [7]. In this study, 4 week old *Alkbh4^L/L^ CreEsr* mice were treated with tamoxifen to delete *Alkbh4* (designated *Alkbh4^Δ/Δ^*). *Alkbh4^L/L^* and *Alkbh4^+/^* ^+^ *CreEsr* mice were used as controls and exposed to the same treatment as *Alkbh4^L/L^ CreEsr* mice (Figure S1 A,B,C). The body weight gain of *Alkbh4^Δ/Δ^* mice was not altered compared to control mice (Figure S2A). The testes of 2 weeks-induced *Alkbh4^Δ/Δ^* mice were significantly smaller than in *Alkbh4^L/L^* and *Alkbh4^+/^* ^+^ *CreEsr* mice and in *Alkbh4^Δ/Δ^* mice treated with tamoxifen for 1 week (Figure 1A). Despite ablation of the *Alkbh4* gene (Figure S1B), the other organs appeared normal at macroscopic inspection. The weight of the spleen was not affected by the depletion of ALKBH4 (Figure S2B). The reduced sizes of testes in *Alkbh4^Δ/Δ^* mice lead us to examine the testicular histology in detail. The diameter of the seminiferous tubuli was significantly reduced in *Alkbh4^Δ/Δ^* mice (Figure 1B, right panel). We observed degeneration of the germinal epithelium marked by loss of germ cells, disruption of the organized germ cell layers, and luminal dislocation of primary spermatocytes (Figure 1C, right panel).

### Increased germ cell apoptosis upon ALKBH4 depletion in mice

**Figure 2:**
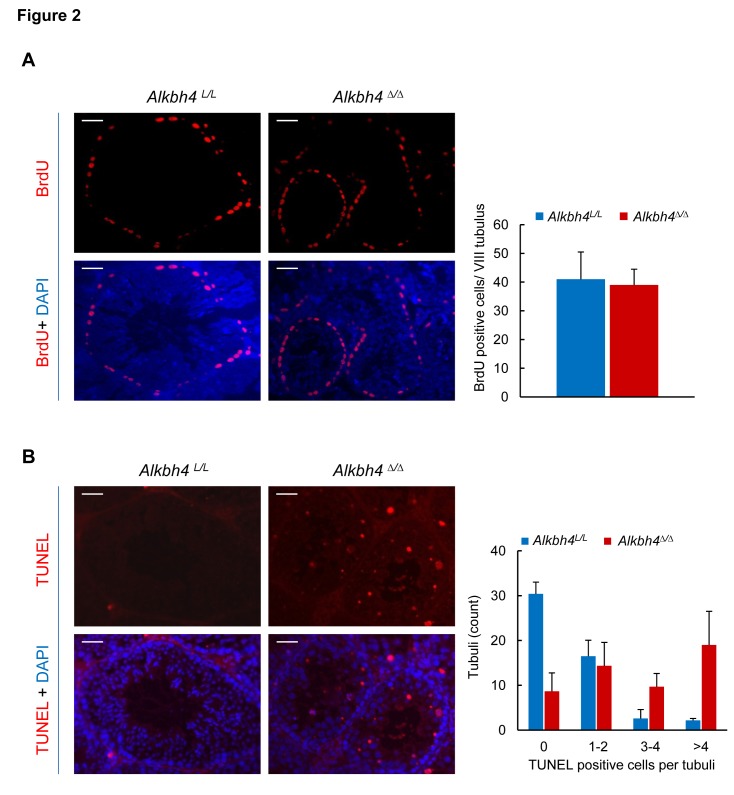
Maintained DNA synthesis and increased apoptosis in testes of *Alkbh4^Δ/Δ^* mice. A Left panel, epifluorescence microscopy of BrdU-labelled testicular sections in 6 weeks old mice treated with tamoxifen for 2 weeks, indicating comparable density of S-phase cells in control and *Alkbh4^Δ/Δ^* mice. DNA was visualized by DAPI staining. Scale bars, 50 µm. Right panel, quantification of the increased BrdU positive cells/stage VIII tubulus in sections (n = 10 tubuli/animal, 2 animals/genotype) in testes of *Alkbh4^Δ/Δ^* compared to *Alkbh4^L/L^* mice. All data expressed as means ± SEM. B Left panel, epifluorescence microscopy of TUNEL-stained testicular sections in 6 weeks old mice treated with tamoxifen for 2 weeks. TUNEL identifies increased proportion of apoptotic germ cells in *Alkbh4^Δ/Δ^* mice compared to control (*Alkbh4^L/L^*). DNA was counterstained with DAPI. Scale bars, 50 µm. Right panel, quantification of the increased number of tubuli with TUNEL positive cells/tubulus in sections (n = 50 tubuli/animal, 3 animals/genotype) in testes of *Alkbh4^Δ/Δ^* compared to *Alkbh4^L/L^* mice. All data expressed as means ± SEM.

To assess whether the reduced tubular diameter and loss of germinal epithelium were associated with either a reduced entry of cells in spermatogenesis or increased cell death, we performed a 2 hour-pulse of 5-bromo-2′-deoxyuridine (BrdU)-incorporation and terminal deoxynucleotidyl transferase (TdT)-mediated dUTP nick end labeling (TUNEL) experiments. The amount of preleptotene spermatocytes, which were identified in stage VII–VIII tubuli by their basal localization and increased BrdU labeling indicative of DNA synthesis, was similar among the control and *Alkbh4* ***^Δ/Δ^*** testis (Figure 2A). The amount of tubuli with a high number of TUNEL positive nuclei was significantly increased (p<0.001) in *Alkbh4* ***^Δ/Δ^*** testes compared to controls (Figure 2B). Apoptotic nuclei were most prevalent in the primary spermatocyte layer of stage VI–XII tubuli, indicating loss of pachytene spermatocytes (Figure 2B).

### Stage-specific loss of testicular germ cells in *Alkbh4^Δ/Δ^* mice

**Figure 3:**
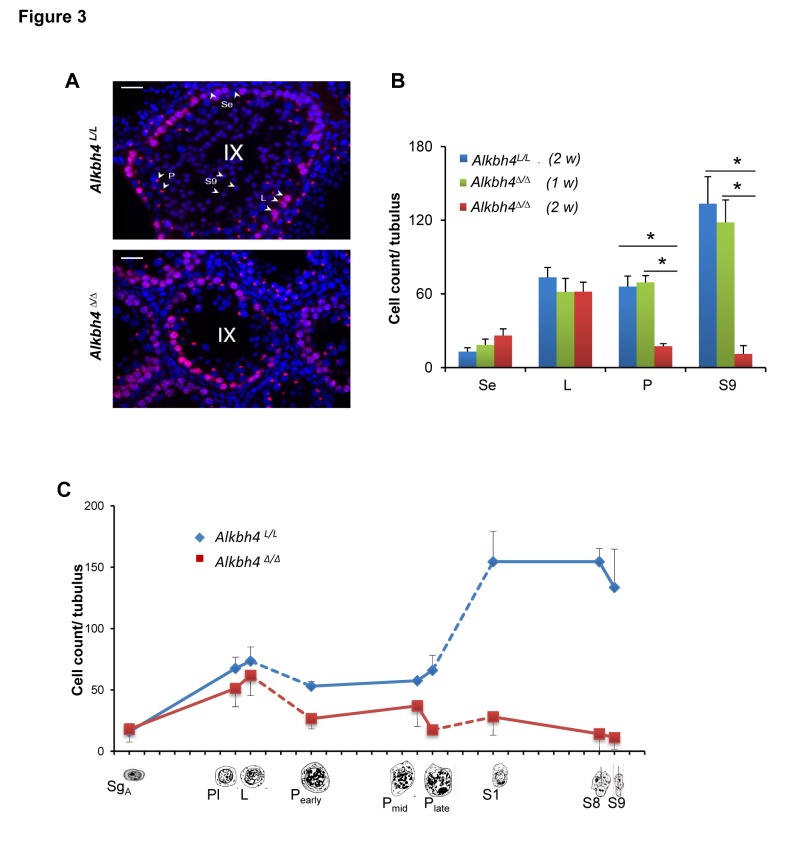
Stage-specific arrest of spermatogenesis in *Alkbh4* ^Δ/Δ^ mice. A Immunofluoresence labeling of histological sections for anti-γH2A.X (red) with DAPI (blue) counterstain, where Sertoli cells (Se), leptotene (L) and pachytene (P) spermatocytes and elongating spermatids (S9) can be distinguished. Scale bar, 20 µm. B Mean cell density per cross-section of stage IX (n>10 tubuli per histological section, total of 6 sections) from control (*Alkbh4^L/L^) and Alkbh4^Δ/Δ^* mice treated with tamoxifen for one or two weeks shows severe depletion of pachytene spermatocytes and S9 elongating spermatids after 2 weeks of tamoxifen treatment in *Alkbh4^Δ/Δ^* mice. Statistical analysis was performed using the non-paired, two-tailed Student's test: *p<0.05. C Mean cell density at selected stages (I, VIII, IX) of spermatogenesis in *Alkbh4^Δ/Δ^* mice indicates depletion of cells at pachynema and all subsequent stages. Dashed lines indicate cell types in tubular stages not counted (late leptonema to early pachynema, late pachynema to round spermatid S1).

The stages of meiotic prophase were assessed in sections immunolabeled for **γ**H2Ax and counterstained with DAPI (Figure 3A). In stage IX tubuli, severe depletion of pachytene spermatocytes and spermatids (S9) was observed in *Alkbh4* ***^Δ/Δ^*** mice after 2 weeks of tamoxifen treatment compared to tamoxifen-treated *Alkbh4* ***^L/L^*** and *Alkbh4* ***^Δ/Δ^*** mice treated with tamoxifen for only 1 week (n>10 tubuli per section, 6 sections per group). The absolute amount of leptotene spermatocytes was unchanged (Figure 3B). The absolute number of Sertoli cells was marginally increased in *Alkbh4* ***^Δ/Δ^*** tubuli (Figure 3B), but increased proliferation of these cells was not observed in BrdU labelling experiments (data not shown).

Comprehensive assessment of germ cell density in *Alkbh4* ***^Δ/Δ^*** and tamoxifen-treated *Alkbh4* ***^L/L^*** mice revealed comparable number of premeiotic spermatogonia, preleptotene and leptotene spermatocytes. Severe lack of pachytene spermatocytes was noted in *Alkbh4* ***^Δ/Δ^*** mice, especially at the late pachytene stage, and depletion of subsequent meiotic stages was apparent (Figure 3C). In normal testis, the average ratio of spermatocytes to round and elongated spermatids is approximately 1∶2∶2 [21]. This ratio was around 1∶1∶1 in ALKBH4-depleted testis (Figure 3C).This shift in the ratio indicates a further loss of germ cells around or during the meiotic divisions, because otherwise the remaining spermatocytes in the ALKBH4-deficient testis should give rise to approximately double the amount of round and elongated spermatids. Careful examination of sectioned stage XII tubuli, where the meiotic divisions take place, did not reveal consistently abnormal or asymmetric metaphases. Metaphase stage cells isolated from stage XII tubuli using the trans-illumination method were also labelled with anti-β-tubulin to visualize the metaphase spindle, but consistent morphological defects were not observed (data not shown). However, this might be due to inefficient Cre recombinase induction in some of the cells.

### Disordered synaptonemal complex in ALKBH4-deficient pachytene spermatocytes

Synaptonemal complex protein 3 (SYCP3) is one of the major components in the synaptonemal complex (SC). This meiosis-specific protein is essential for the axial element assembly along the chromosomes and it is necessary for accurate synapsis formation (reviewed in [22]).

**Figure 4:**
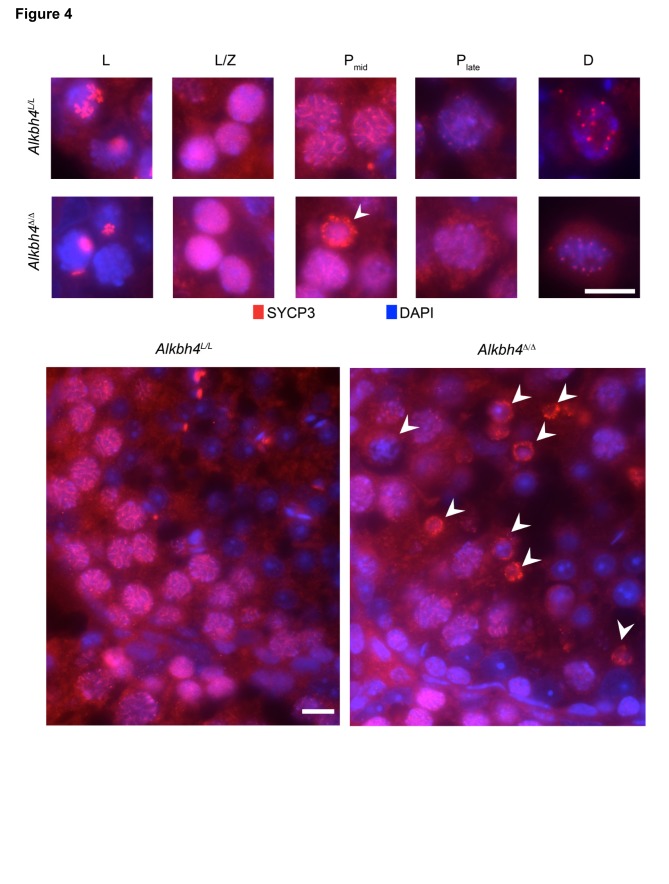
Pachytene spermatocytes in *Alkbh4* ^Δ/Δ^ mice fail to maintain synaptonemal complex. Upper panels, immunofluorescence labeling of histological sections for SYCP3 (red) and DAPI (blue counterstain) allows distinguishing leptotene (L) spermatocytes, cells at leptonema-zygonema transition (L/Z), as well as pachytene (P) and diakinesis-stage (D) spermatocytes by the characteristic development of the synaptonemal complex (SC). Lower panels, among pachytene spermatocytes in *Alkbh4^Δ/Δ^* mice, cells with disordered SC can be observed (arrows). Scale bars, 10 µm.

In *Alkbh4^L/L^* leptonema, intranuclear aggregates of SYCP3 were detected (Figure 4). In the leptonema/zygonema transition, the aggregates decreased in size, and smaller foci/short stretches of SYCP3 in the SCs appeared. The mature SCs, formed along the entire chromosome axes in pachynema, were apparent as longer SYCP3-positive threads. At the diakinesis stage of meiosis, SYCP3 was removed from chromosome arms, but was maintained in smaller foci (corresponding to paired centromeres). These observations are in agreement with previously reported SYCP3-staining pattern in mouse spermatocytes [23], [24].

In *Alkbh4^Δ/Δ^* mice (tamoxifen-treated for 2 weeks), the SCs developed normally in leptotene and leptotene/zygotene stages, and occasionally, spermatocytes progressed beyond the diakinesis stage (Figure 4). In pachytene stage, an increased proportion of spermatocytes lost the organized SCs, which appeared as shorter SYCP3-positive segments, diffuse cytoplasmatic SYCP3 signal and weaker SYCP3 signal in the SCs. Moreover, in a fraction of the affected spermatocytes we observed SYCP3 staining as condensed protein fragments under the nuclear membrane. The nuclei of these cells were shrunken and the cells dislocated towards the lumen, indicative of cell death. Similar observations were made using spread pachytene spermatocytes derived from *Alkbh4^Δ/Δ^* mice, which occasionally lacked a mature synaptonemal complex (Figure S3). Furthermore, staining for the synaptonemal complex component SYCP1 (Figure S4) also revealed the presence of pachytene spermatocytes with disorganized complex in *Alkbh4^Δ/Δ^* mice.

### ALKBH4 localizes to the nucleolar structures in spermatogenic and Sertoli cells

**Figure 5:**
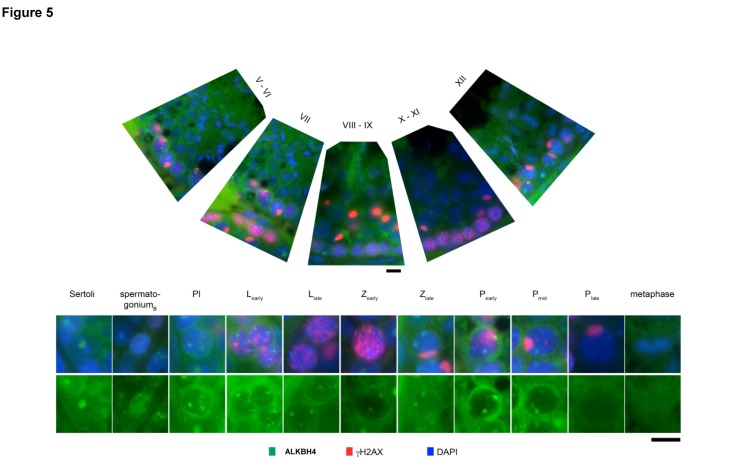
Nuclear localization of ALKBH4 in seminiferous tubuli. Upper panels, overview of the spermatogenic cycle in mouse testis with immunofluorescence labeling for ALKBH4 (green), γH2A.x (red) and DNA (DAPI, blue). Lower panels, ALKBH4 shows nucleolar localization in Sertoli cells. During the meiotic prophase, ALKBH4 is distributed in diffuse nuclear patches throughout pre-leptotene (Pl), leptotene (L), and zygotene (Z) stages, and disappears towards late pachynema (P). Scale bar, 10 µm.

Selective loss of pachytene spermatocytes in *Alkbh4^Δ/Δ^* mice led us to examine the expression of ALKBH4 protein throughout spermatogenesis (Figure 5, upper panel). We have previously characterized the localization of ALKBH4 in the cleavage furrow during cytokinesis [7]. We were not able to assess a detailed cytoplasmic localization pattern for ALKBH4 in the testis sections studied in this report. However, in the nuclei of Sertoli cells, spermatogonia, and zygotene and pachytene spermatocytes of wild-type mice, ALKBH4 localized to distinct structures in euchromatin, often in close association with heterochromatin. In Sertoli cells, a single ALKBH4-rich structure was observed in each nucleus. In spermatogonia, ALKBH4 was distributed in fine granular patches under the nuclear envelope. In preleptotene (Pl) and early leptotene (L_early_) spermatocytes, ALKBH4 formed 3 to 8 diffuse threads and patches per nucleus. During the late leptotene (L_late_) to the mid-pachytene (P_mid_) stages, the number of ALKBH4-rich patches decreased and localized in the periphery of the nucleus. Expression of ALKBH4 diminished during the late pachynema (P_late_) and diakinesis (Figure 5, lower panel). In *Alkbh4* ***^Δ/Δ^*** mice treated with tamoxifen for one and two weeks, an overall decrease of ALKBH4-specific immunofluorescence was observed compared to the *Alkbh4^L/L^* samples (Figure S5).

We speculated if the ALKBH4 positive structures in the nuclei could be related to nucleolar compartments, as has been reported previously for ectopically expressed ALKBH4 in cell culture studies [9]. Co-immunostaining with anti-ALKBH4 and anti-fibrillarin, a nucleolar marker, verified the localization of ALKBH4 in the nucleoli (Figure S6).

## Discussion

In this study, we used an inducible gene-targeting approach to disrupt the *Alkbh4* gene in mice to reveal its function in a physiological context. The embryonic lethal phenotype of the *Alkbh4^−/−^* mice, failure of cytokinesis and increased cell death after depletion of ALKBH4 have been previously described [7]. Thus, it was surprising that the induced mutant mice were viable with general normal appearance except for disruption of spermatogenesis. This leads us to hypothesize that ALKBH4 might play a distinct role in development, both in embryogenesis and in gametogenesis.

We found that depletion of ALKBH4 in mice does not seem to influence the entry of germ cells into meiosis, but ALKBH4 is required for successful completion of the meiotic prophase. Indeed, spermatogenesis was arrested at the pachytene stage in *Alkbh4* ***^Δ/Δ^*** mice, indicated by a reduced density of pachytene spermatocytes, increased rate of apoptosis, as well as dispersion of the organized synaptonemal complex. The gradual loss of nuclear ALKBH4 towards the late pachytene phase also supports the notion that *Alkbh4* has a specific function during the prophase of meiosis.

Although the molecular defects leading to meiotic arrest at pachynema in *Alkbh4* ***^Δ/Δ^*** mice are unknown, the disordered axial elements seen after depletion of ALKBH4 leads us to propose that the loss of cells at the pachytene stage is related to a failure of SC formation, with subsequent activation of pachytene checkpoint control mechanisms and apoptosis [25]. A fraction of ALKBH4-depleted spermatocytes did nevertheless complete meiosis, but these surviving cells gave rise to a lower than expected number of haploid spermatids. Although we cannot exclude the possibility that the incomplete inducible knockout after Cre recombinase induction is responsible for this effect, the reduction of spermatids may indicate that lack of ALKBH4 also affects spermatogenesis beyond the pachytene stage. We have previously described ALKBH4 as a modulator of specific actin-myosin dynamics in the cytoplasm, via regulation of the K84me1-level in actin in cell culture [7]. Interestingly, in this study we found ALKBH4 to localize to distinct euchromatic aggregates/patches in the nucleus of spermatogenic cells and in the nucleolus of Sertoli cells, which may support the involvement of ALKBH4 in regulation of specific actin dynamics in the nucleus. These dynamics may be required for normal development of pre- and post-meiotic germ cells, via regulation of the K84me1 modification. In the cell nucleus, actin interacts with many different proteins involved in chromatin structure and function [26], [27], transcription initiation and elongation [28]–[30], and RNA processing [31], [32]. In the early stages of premeiotic spermatocytes (preleptotene and mid-leptotene) ALKBH4 is found in several threads and patches in the nucleus. From late leptonema to mid-pachynema, the number of patches decreases. Interestingly, ALKBH4 does not seem to be present as aggregates in the nuclei of late pachytene and metaphase cell types. Accordingly, we found ALKBH4 to colocalize with the nucleolar marker fibrillarin in spermatogenic cells indicating, that these structures are nucleolar organizing regions (NORs). NORs are engaged in ribosome biogenesis [33] and found to associated with several autosomal bivalents in meiotic prophase spermatocytes [34]. At mid-pachytene the NORs detach from their autosomal bivalents and associate closely with the XY chromosomal pair (termed the XY-body). The nucleolus associated with the XY pair appears to be transcriptionally inactive [35]. We could not detect aggregates of ALKBH4 near the XY-body in pachytene stage spermatocytes, which also reflects the possibility that ALKBH4 is predominantly present at transcriptionally active sites.

Organization of the chromosomes and timed homologous recombination events during the prophase of meiosis requires a highly organized cell machinery. Actin has been shown to be involved in many nuclear processes in yeast meiocytes, such as pairing of chromosomal homologues, formation of synaptonemal complex and telomeric organization/bundling in zygotene stage cells. There may be a network of both nuclear and cytoplasmic actin interaction in these processes [15], [36], [37]. The role of actin dynamics during mammalian spermatogenesis, however, remains to be explored [17], [38]. Studies of long-range interphase chromosome movements in mammalian somatic cells show dependency on nuclear actin and myosin [39]. In mammalian primary spermatocytes, actin may also play a part in the process of homologous chromosome pairing and formation of the synaptonemal complex. It is possible that many of the same processes could relate to mammalian meiotic cells, with ALKBH4 as an important modulator.

In conclusion, we report a role of ALKBH4 in spermatogenesis as an important factor for the development of premeiotic I stage cells and postmeiotic cells. Defects in gametogenesis are a major source of human reproductive failure, and the results presented here could be important in the understanding of mechanisms lying behind infertility.

## Materials and Methods

### Ethics

All experimental procedures were approved by the Norwegian Animal Research Authority in accordance with institutional rules and national legislation. Mice were housed in the minimal disease unit under barrier conditions.

### *Alkbh4^L/L^ Cre* mice and controls

We have described the DNA sequence in the *Alkbh4* allele flanked by *LoxP* (*Alkbh4^L/L^*) previously [7]. *Alkbh4^L/L^* mice were mated with *CreEsr* transgenic mice [40] (Jackson Laboratories, West Grove, PA) to create the inducible knockout genotype *Alkbh4^L/L^ CreEsr*.

Deletion of *Alkbh4* (herein termed *Alkbh4^Δ/Δ^*) in *Alkbh4^L/L^ Cre-ER* mice was achieved by daily i.p. injection of tamoxifen (Sigma) (1 mg/20 g bodyweight) in corn oil for 7 or 14 consecutive days. Injections started in 4-week-old mice (Figure S1 a). *Alkbh4^L/L^* mice injected with tamoxifen were used as controls. Tamoxifen-injected *Alkbh4^+/+^ CreEsr* mice were used as additional control to assess the effect of the activated Cre recombinase. The genotype was determined by standard PCR methods as described previously [7].

### Histopathology

Testes were fixed in 10% neutral buffered formalin and embedded in paraffin. Sections (4 µm) were fixed on slides, deparafinized and rehydrated in 100-70% ethanol. The slides were stained with hematoxylin and eosin (Richard-Allan Scientific) according to standard protocols. After dehydration, the slides were washed in Clear Rite 3 and mounted using Mounting medium 4111 (Richard-Allan Scientific). Images of stained sections were acquired using AxioCam ICc1 camera on an Axio Observer.Z1 microscope (Carl Zeiss).

### Histological analysis

Tissues were fixed in 10% neutral buffered formalin and embedded in paraffin. For histology, tissue sections were stained with hematoxylin and eosin (H&E) according to standard protocols and examined by light microscopy. For immunohistochemistry, tissue sections were deparaffinized, rehydrated, and subjected to heat-induced epitope retrieval in Tris-EDTA buffer (10 mM Tris, 1 mM EDTA, pH 9). The sections were permeabilized with 0.1% Triton-X 100 in TBS-T (TBS with 0.1% Tween) for 15 min at room temperature and blocked with 0.5% BSA, 0.5% goat serum in TBST for 1 hour at room temperature. Slides were incubated with polyclonal rabbit anti ALKBH4 (1∶500, #282 [7] overnight at 4°C, followed by incubation with 0.3% H_2_O_2_ for 10 minutes, incubation with secondary antibody, and staining according to the EnVision+HRP kit (DAKO, K4010). For immunofluorescence, tissue section were prepared as described above and permeabilized with 0.5% triton-x 100 in TBST for 10 min at room temperature and blocked with 0.5% dried milk +0.5% serum (normal serum from the same species as secondary antibodies) in TBST for 1 hour at room temperature. Slides were incubated with primary antibody for 1 hour or overnight at 4°C before incubation with secondary antibody. DAPI (1 µg/ml) was used as counterstain and Mowiol 4–88 (Polysciences) was used for mounting. Primary antibodies used: goat-anti yH2A.x (1∶500, Abcam), rabbit polyclonal anti-ALKBH4 (1∶1000,#282), anti-SYCP3 (20 mg/ml, LifeSpan Biosciences), anti-fibrillarin (1∶100, Santa Cruz), anti-SYCP1 (1∶100, Abcam). Secondary antibodies: donkey-anti-goat-Alexa 488/594 (1∶500, Invitrogen), goat anti-rabbit-Alexa 488 (1∶500, Invitrogen). Images of stained sections were acquired using AxioCam MRRev3 camera on an Axio Observer.Z1 microscope (Carl Zeiss).

### BrdU labeling

To observe BrdU (5-bromo-2-deoxyuridine) incorporation in testis, mice were injected i.p with 1 mg BrdU (Sigma) and sacrificed after 2 hours. Staining of testis sections were done according to protocol described (BrdU protocol, Abcam). Rat anti BrdU (1∶100, Abcam) was used as primary antibody, and goat anti-rat Alexa594 (1∶500, Invitrogen) was used as secondary antibody. Sections were counterstained with DAPI and mounted with Mowiol. Images of stained sections were acquired using AxioCam MRRev3 camera on an Axio Observer.Z1 microscope (Carl Zeiss).

### Apoptosis detection

The *In Situ* Cell Death Detection Kit, TMR red, (Roche) was used for TUNEL labeling of testis sections according to protocol described (Roche). Sections were counterstained with DAPI and mounted with Mowiol. Images of stained sections were acquired using AxioCam MRRev3 camera on an Axio Observer.Z1 microscope (Carl Zeiss).

### Immunoblotting

Testes were collected and snap frozen in liquid nitrogen and stored at −80°C until used. Whole protein extracts from testis were made by homogenizing and lysing tissue in RIPA (RadioImmunoPrecipitation Assay) buffer (50 mM Tris HCl pH 8, 150 mM NaCl, 1% NP-40, 0.5% sodium deoxycholate, 0.1% SDS) added complete protease inhibitor cocktail (Roche) with a bead-based homogenization system (Lysing Matrix, MP Biomedicals).Protein concentration was determined using the Bradford assay. Equal amounts of total protein were separated by SDS-PAGE, transferred to a PVDF membrane and immunoblotted with the following primary antibodies: rabbit anti-ALKBH4 (#282, 1∶500), goat anti-α-tubulin (1∶8000, Sigma). HRP-labeled secondary antibodies were detected using the SuperSignal West Dura substrate (Thermo Scientific) and ChemiDoc XRS+ System (Bio-Rad).

### Statistical analysis

Statistical analysis was performed using GraphPad Prism 5.0 software. Unpaired two-tailed T-tests were used to compare two groups with normal distributed data. The Mann-Whitney-U test was performed for the analysis of ordinally scaled data. A p-value of<0.05 was considered statistically significant.

## Supporting Information

Figure S1 **Outline of tamoxifen treatment and suppression of** ***Alkbh4*** **detected by PCR and Western blot.** Schematic outline of TAM treatment in of *Alkbh4^L/L^ CreEsr* and control mice day 0 to 14 with indicated time-points for sampling (A). Cre-mediated recombination of the LoxP-flanked DNA sequence in selected organs of *Alkbh4^Δ/Δ^* mice after 2 weeks of tamoxifen treatment detected by PCR (B). Depletion of ALKBH4 in whole-testis extracts of *Alkbh4^Δ/Δ^* mice after 2 weeks of tamoxifen treatment detected by Western blot (C).
(TIF)

Figure S2 **Depletion of ALKBH4 does not alter weight gain and relative weight of spleen in mice.** *Alkbh4^Δ/Δ^* mice have similar weight gain during tamoxifen treatment as control mice. Weight gain (gram) in 6 weeks old male and female mice shown after treatment with tamoxifen for two weeks (Male: *CreEsr*, n = 5; *Alkbh4^L/L^*, n = 9; *Alkbh4^Δ/Δ^*, n = 9. Female: *CreEsr*, n/a; *Alkbh4^L/L^*, n = 11; *Alkbh4^Δ/Δ^*, n = 12). Data are expressed as means ± SD (A). Weight of spleen relative to body weight is not affected by loss of ALKBH4. (*CreEsr*, 3; *Alkbh4^L/L^*, n = 4; *Alkbh4^Δ/Δ^*, n = 6). Data are expressed as means ± SD (B).
(TIF)

Figure S3 **Disorganization of the synaptonemal complex in pachytene spermatocytes of** ***Alkbh4^Δ/Δ^*** **mice.** After dissection of seminiferous tubuli, spermatocytes were spread on glass slides under a coverslip, snap-frozen on liquid nitrogen, and fixed in cold ethanol. The slides were screened for appropriate spreading of cells and signs of nuclear swelling, and suitable specimens were probed with anti-SYPC3 antibody (red), the CREST antiserum (green) that marks centromeres, and appropriate. Pachytene spermatocytes derived from *Alkbh4^Δ/Δ^* mice occasionally lacked an synaptonemal complex organized along the chromosome axes. Scale bar, 10 µm.
(PDF)

Figure S4 **Defects of synaptonemal complex organization in pachytene spermatocytes of** ***Alkbh4^Δ/Δ^*** **mice.** Histological sections of testes from the control *Alkbh4^L/L^* and the *Alkbh4^Δ/Δ^* mice were probed with antibodies against SYCP1 (*green*), γH2Ax (*red*). Tubular cross-sections corresponding to stage VIII of the spermatogenic cycle were identified by the presence of pre-leptotene spermatocytes, located basally in the tubuli and displaying fine dispersed nuclear γH2Ax signal; pachytene spermatocytes with mature full-length synaptonemal complexes, distinct sex body, and lack of dispersed γH2Ax signal; where present, spermatids corresponding to stage 7 nuclear morphology with DAPI (*blue*) stain. In *Alkbh4^Δ/Δ^* mice, occasional, luminally dislocated pachytene spermatocytes were observed lacking an organized synaptonemal complex (arrows). Scale bar, 10 µm.
(PDF)

Figure S5 **Reduced expression of ALKBH4 in** ***Alkbh4^Δ/Δ^ mice*** **.** Immunohistochemical labelling of testis sections show lower levels of ALKBH4 in testis from *Alkbh4^Δ/Δ^* mice treated with tamoxifen for 1 week. Left panels show sections of *Alkbh4^L/L^* testes. Right panels show sections of *Alkbh4^Δ/Δ^* testes Scale bars, 10 µm (A). Immunofluorescent staining of ALKBH4 in *Alkbh4^L/L^* (left panel) and *Alkbh4^Δ/Δ^* (right panel) testes from mice treated with tamoxifen for 2 weeks Scale bars, 10 µm (B).
(PDF)

Figure S6 **ALKBH4 and fibrillarin in Sertoli cells.** Immunofluorescent labeling of testis section with anti-ALKBH4 (green) and anti-fibrillarin (red) show nucleolar localization. DNA counterstained with DAPI. Scale bar, 5 µm.
(PDF)
