# Supplementary figures and images for: ALKBH4 Depletion in Mice Leads to Spermatogenic Defects
Source: PLoS One. 2014 Aug 25;9(8):e105113. doi: 10.1371/journal.pone.0105113 (PMC4143218; doi:10.1371/journal.pone.0105113)

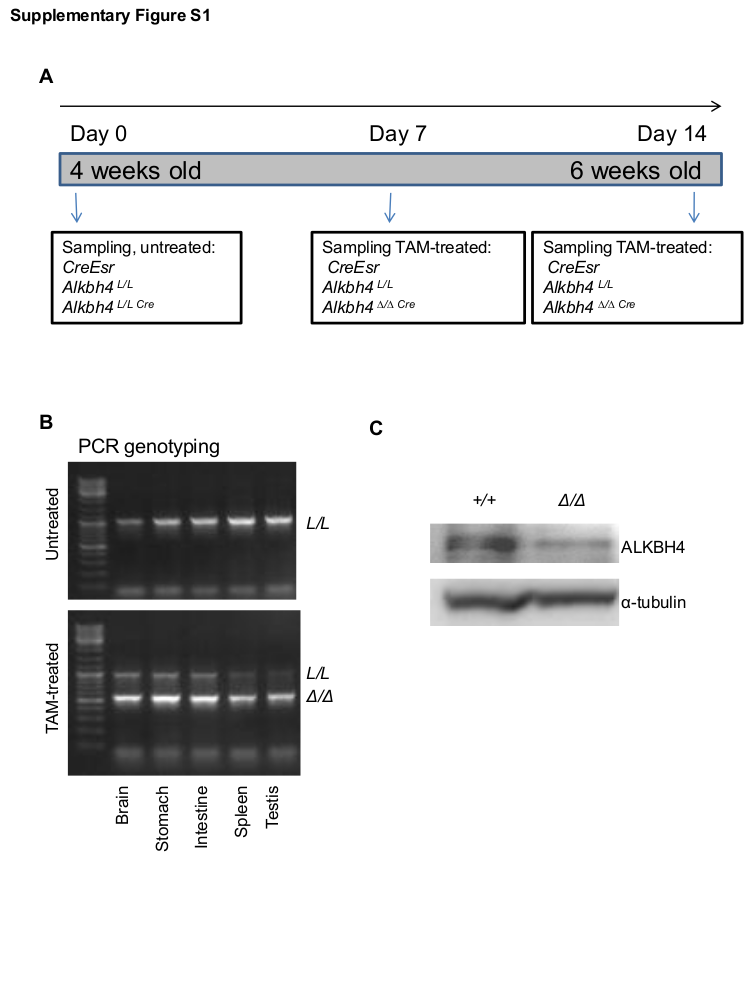

Supplement: Figure S1 — Outline of tamoxifen treatment and suppression of Alkbh4 detected by PCR and Western blot. Schematic outline of TAM treatment in of Alkbh4L/L CreEsr and control mice day 0 to 14 with indicated time-points for sampling (A). Cre-mediated recombination of the LoxP-flanked DNA sequence in selected organs of Alkbh4Δ/Δ mice after 2 weeks of tamoxifen treatment detected by PCR (B). Depletion of ALKBH4 in whole-testis extracts of Alkbh4Δ/Δ mice after 2 weeks of tamoxifen treatment detected by Western blot (C). (TIF) [file pone.0105113.s001.tif]

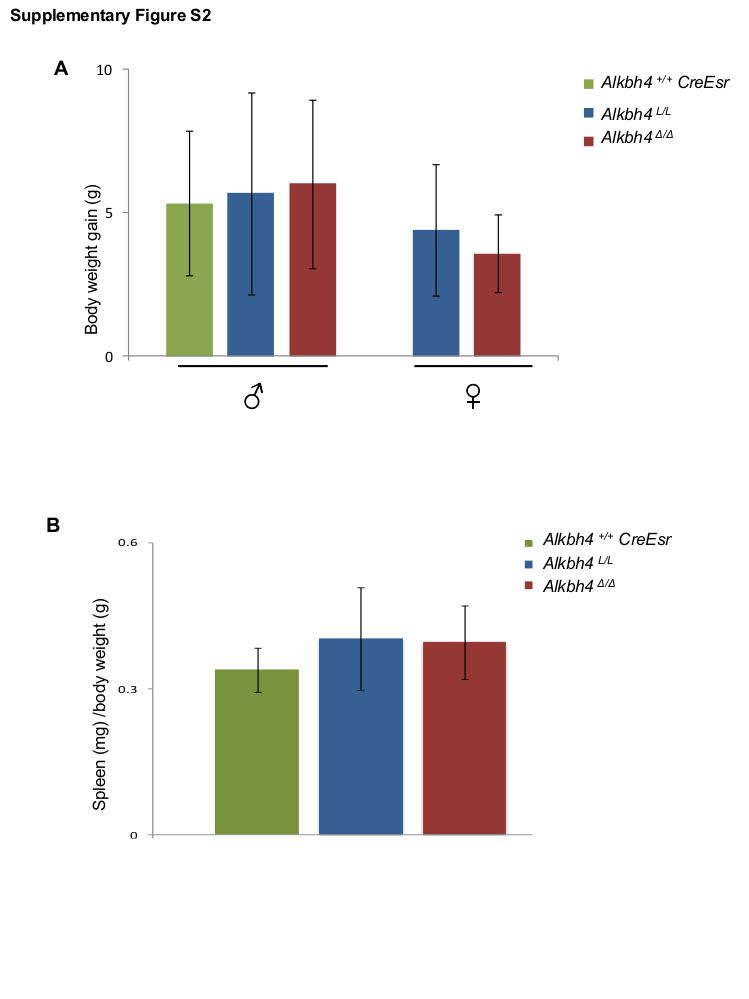

Supplement: Figure S2 — Depletion of ALKBH4 does not alter weight gain and relative weight of spleen in mice. Alkbh4Δ/Δ mice have similar weight gain during tamoxifen treatment as control mice. Weight gain (gram) in 6 weeks old male and female mice shown after treatment with tamoxifen for two weeks (Male: CreEsr, n = 5; Alkbh4L/L, n = 9; Alkbh4Δ/Δ, n = 9. Female: CreEsr, n/a; Alkbh4L/L, n = 11; Alkbh4Δ/Δ, n = 12). Data are expressed as means ± SD (A). Weight of spleen relative to body weight is not affected by loss of ALKBH4. (CreEsr, 3; Alkbh4L/L, n = 4; Alkbh4Δ/Δ, n = 6). Data are expressed as means ± SD (B). (TIF) [file pone.0105113.s002.tif]

# Supplementary Figure S3

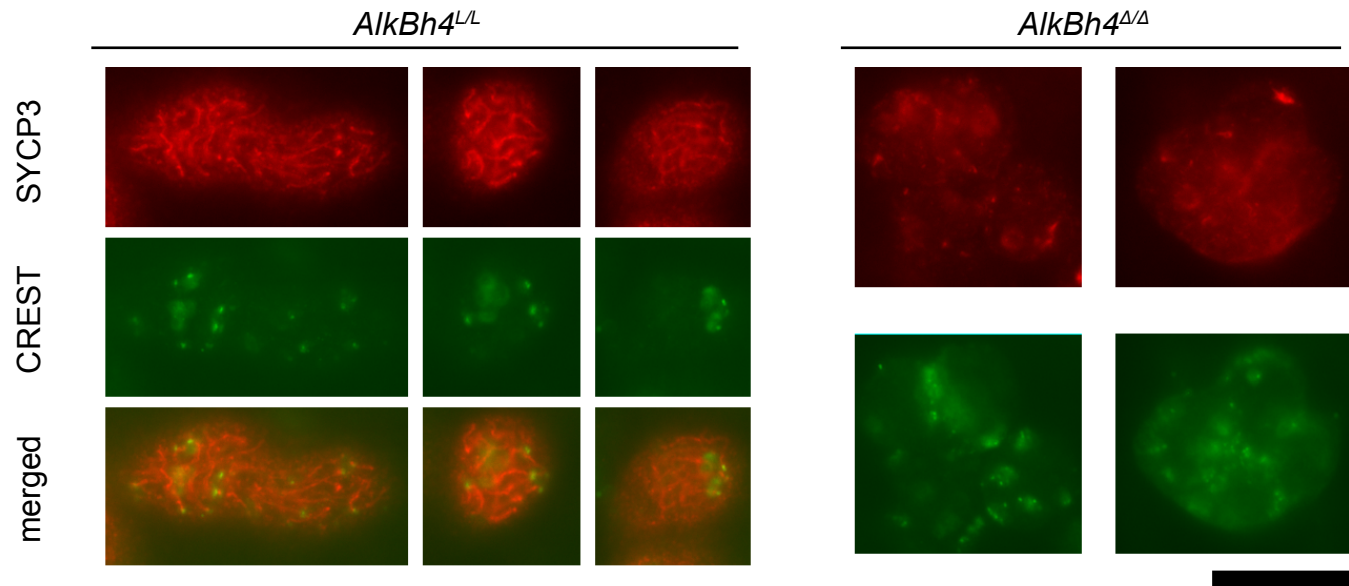

Supplement: Figure S3 — Disorganization of the synaptonemal complex in pachytene spermatocytes of Alkbh4Δ/Δ mice. After dissection of seminiferous tubuli, spermatocytes were spread on glass slides under a coverslip, snap-frozen on liquid nitrogen, and fixed in cold ethanol. The slides were screened for appropriate spreading of cells and signs of nuclear swelling, and suitable specimens were probed with anti-SYPC3 antibody (red), the CREST antiserum (green) that marks centromeres, and appropriate. Pachytene spermatocytes derived from Alkbh4Δ/Δ mice occasionally lacked an synaptonemal complex organized along the chromosome axes. Scale bar, 10 µm. (PDF) [file pone.0105113.s003.pdf]

Supplementary Figure S4

*AlkBh4<sup>L/L</sup>*

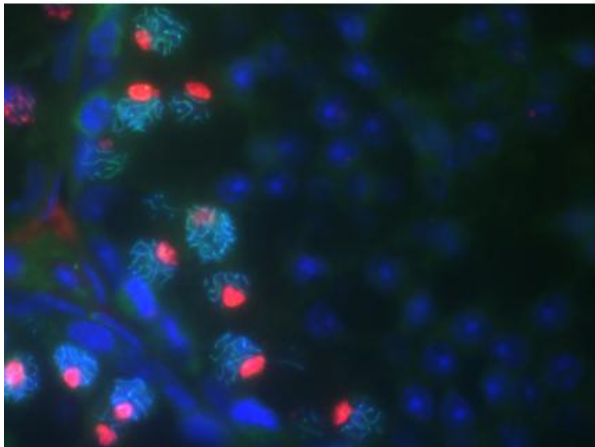

*AlkBh4<sup>Δ/Δ</sup>*

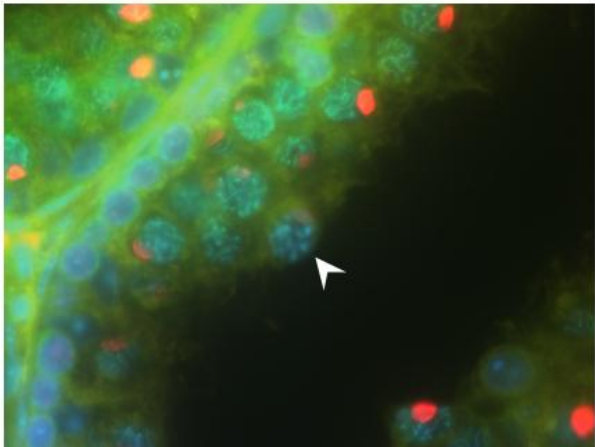

*AlkBh4<sup>Δ/Δ</sup>*

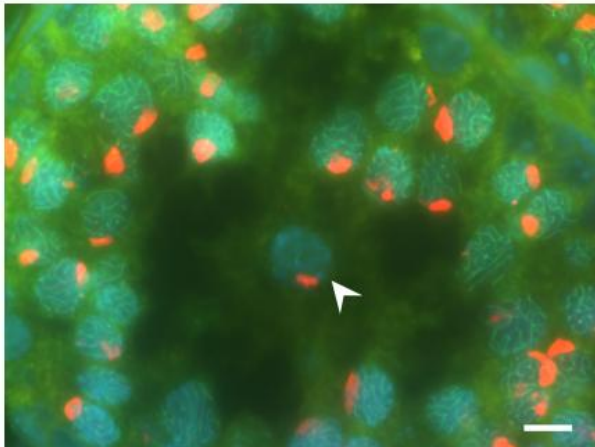

Supplement: Figure S4 — Defects of synaptonemal complex organization in pachytene spermatocytes of Alkbh4Δ/Δ mice. Histological sections of testes from the control Alkbh4L/L and the Alkbh4Δ/Δ mice were probed with antibodies against SYCP1 (green), γH2Ax (red). Tubular cross-sections corresponding to stage VIII of the spermatogenic cycle were identified by the presence of pre-leptotene spermatocytes, located basally in the tubuli and displaying fine dispersed nuclear γH2Ax signal; pachytene spermatocytes with mature full-length synaptonemal complexes, distinct sex body, and lack of dispersed γH2Ax signal; where present, spermatids corresponding to stage 7 nuclear morphology with DAPI (blue) stain. In Alkbh4Δ/Δ mice, occasional, luminally dislocated pachytene spermatocytes were observed lacking an organized synaptonemal complex (arrows). Scale bar, 10 µm. (PDF) [file pone.0105113.s004.pdf]

Supplementary Figure S5

A

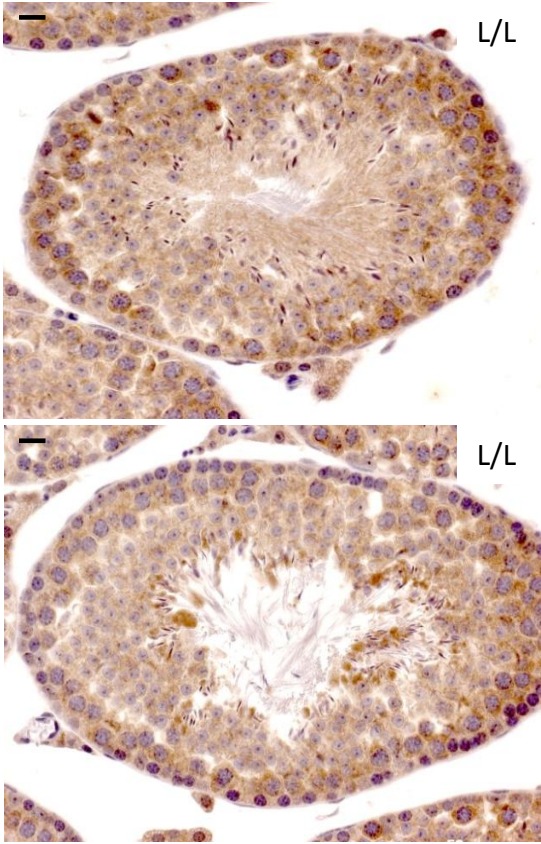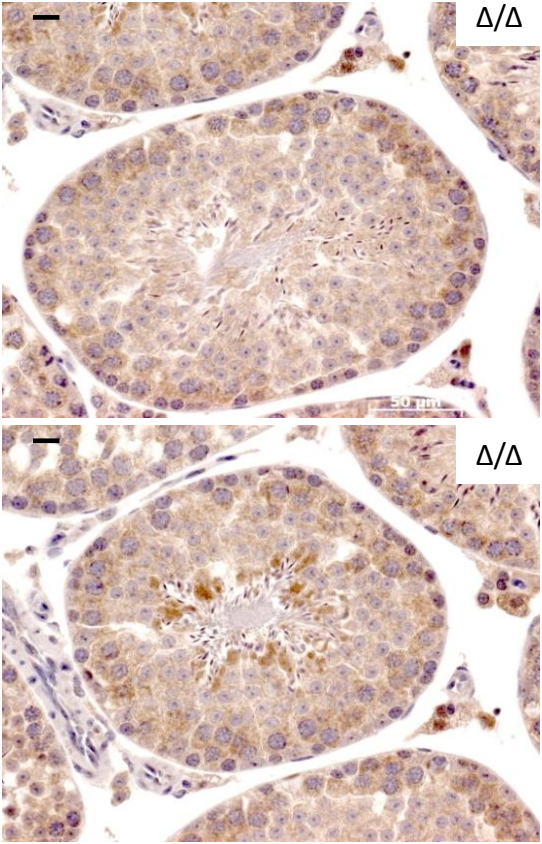

B

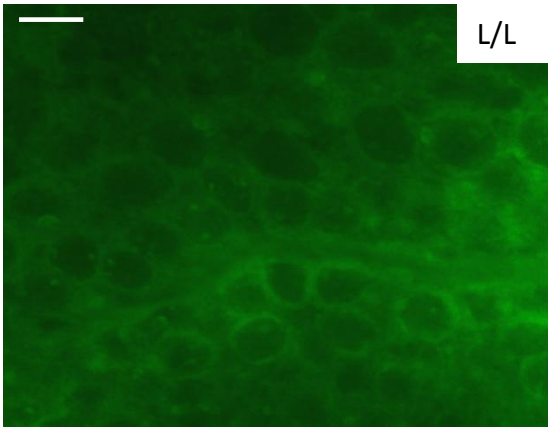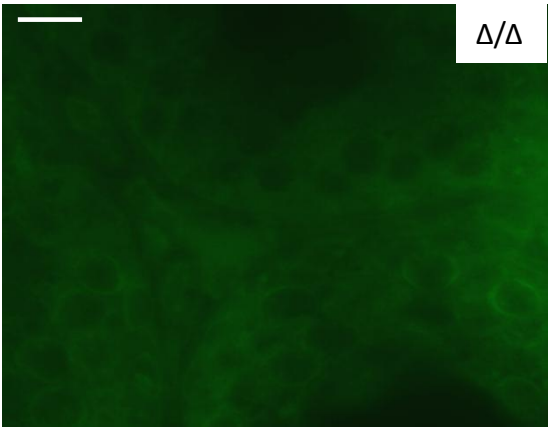

Supplement: Figure S5 — Reduced expression of ALKBH4 in Alkbh4Δ/Δ mice . Immunohistochemical labelling of testis sections show lower levels of ALKBH4 in testis from Alkbh4Δ/Δ mice treated with tamoxifen for 1 week. Left panels show sections of Alkbh4L/L testes. Right panels show sections of Alkbh4Δ/Δ testes Scale bars, 10 µm (A). Immunofluorescent staining of ALKBH4 in Alkbh4L/L (left panel) and Alkbh4Δ/Δ (right panel) testes from mice treated with tamoxifen for 2 weeks Scale bars, 10 µm (B). (PDF) [file pone.0105113.s005.pdf]

Supplementary Figure S6

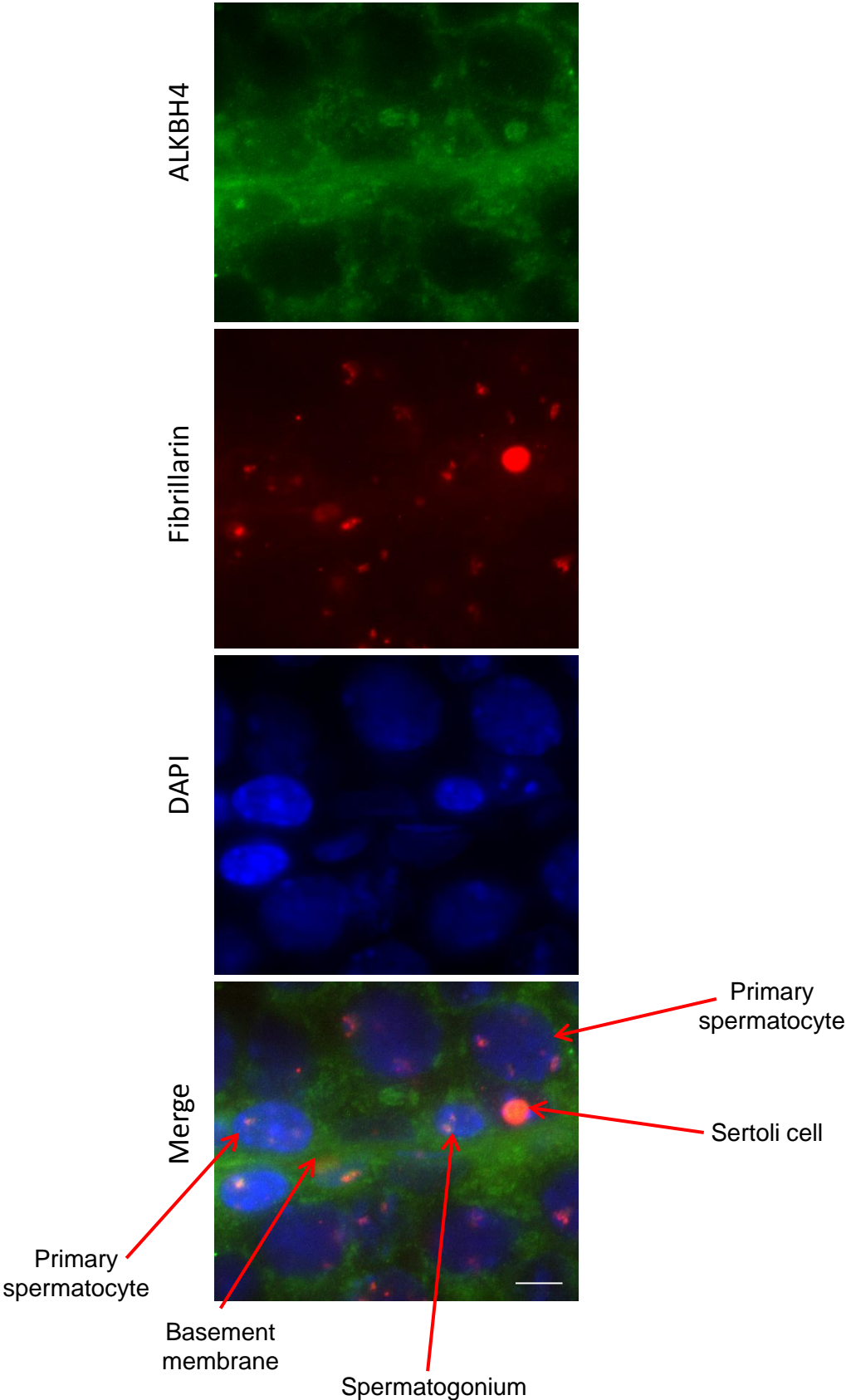

Supplement: Figure S6 — ALKBH4 and fibrillarin in Sertoli cells. Immunofluorescent labeling of testis section with anti-ALKBH4 (green) and anti-fibrillarin (red) show nucleolar localization. DNA counterstained with DAPI. Scale bar, 5 µm. (PDF) [file pone.0105113.s006.pdf]
